# Supplementary material for: Barriers, enablers and acceptability of home-based care following elective total knee or hip replacement at a private hospital: A qualitative study of patient and caregiver perspectives
Source: PLoS One. 2022 Aug 24;17(8):e0273405. doi: 10.1371/journal.pone.0273405 (PMC9401137; doi:10.1371/journal.pone.0273405)
Supplement: S1 Table — (DOCX) [file pone.0273405.s001.docx]

**S1 Table. Patient interview schedule.**

Part one: explore the barriers to, and enablers of hospital at home and rehabilitation at home, in lieu of inpatient care using the Theoretical Domains Framework

*Motivation and goals*

1. Please describe why you prefer/preferred inpatient or home-based care?
2. What do you believe are the benefits and/or disadvantages of inpatient or home-based care

*Social influences*

1. What are the views of your family and/or friends about inpatient and home-based care?
2. How important are these views to you?

*Environmental context and resources*

1. In what ways (if any) do/did inpatient facilities influence your preference for rehabilitation setting? (Prompts if needed e.g., hospital bed, meals, staff, equipment)
2. In what ways (if any) do/did your home influence your preference for rehabilitation setting? (Prompts if needed, e.g., living upstairs)
3. Are there any other factors that influenced your preference for rehabilitation? (Prompt surgeon/other health care provider, benefits such as outcome, medical support, insurance, other support)

*Knowledge*

1. Do you know and can describe what care options (are/were) available to you?

*Beliefs about consequences*

1. What do you believe will happen to you if you are discharged directly home, instead of inpatient rehabilitation? [If preferred home or was discharged home - what do you believe will happen to you if you are discharged home earlier, such as day 3?]

*Emotion*

1. To what extent do/did emotional factors (e.g., worry, fear, anxiety) deter/ed you from having care at home

*Beliefs about capabilities*

1. In what ways could the hospital service make it easy/easier for you to be discharged directly home, instead of inpatient rehabilitation?

*Optimism*

1. How confident are you that care at home will result in a good outcome, just as good as inpatient rehabilitation?

Part two: explore components of home services that may be acceptable to privately insured patients and their caregivers

1. To assist you and/or other patients to be discharged directly home instead of inpatient rehabilitation, what health care information would you need to know? How would this information be best delivered to you? When? By whom?
2. What health professional support might you need at home? (Prompt for specific health care professionals, including interventions and intensity)
   - 1. Rehabilitation specialist or GP? If yes, what medical care would you need at home? (Prompt pain management). Any other specific medical reasons you need a doctor at home? How many visits per week? How many weeks?
     2. Nursing? If yes, what nursing care would you need at home? (Prompt pain or wound management), any other specific reasons you need a nurse at home? How many visits per week? How many weeks?
     3. Physiotherapy? If yes, what physiotherapy care would you need at home? (Prompt exercise therapy, exercises progressions) Any other specific reasons you need a physiotherapist at home? How many visits per week? How many weeks?
3. What other services or supports might you need? (Prompt domestic services such as assistance with transport, shopping, meals, etc.)
   - 1. Transport services? If yes, where to? (Prompt to/from hospital, grocery shopping, attend medical or physiotherapy appointments) What transport services (Prompt Taxi, Uber, other). How often? How many weeks?
     2. Meals services? If yes, what type of meals? (Prompt meals on wheels, Uber eats) How many meals per day? How many weeks?
     3. Cleaning service? If yes, what type of cleaning? How many services per week? How many weeks?
     4. Self– care assistance? What type of self-care services? (Prompt Showering) How much self-care per day? How many weeks?
     5. Is there any other services supports you/other patients might need to be discharged directly home instead on inpatient rehabilitation?
