# Supplementary material for: Barriers, enablers and acceptability of home-based care following elective total knee or hip replacement at a private hospital: A qualitative study of patient and caregiver perspectives
Source: PLoS One. 2022 Aug 24;17(8):e0273405. doi: 10.1371/journal.pone.0273405 (PMC9401137; doi:10.1371/journal.pone.0273405)
Supplement: S2 Table — (DOCX) [file pone.0273405.s002.docx]

**S2 Table. Caregiver interview schedule.**

Part one: explore the barriers and enablers for hospital at home and rehabilitation at home, in lieu of inpatient care using the Theoretical Domains Framework

*Motivation and goals*

1. After surgery, what type of activities would or did you provide assistance with for your [patient]?
2. Please describe why you think [patient] **preferred** inpatient care [or care at home] after their surgery? (Prompt benefits such as outcome, medical support, other support, insurance)
3. What do you believe are the benefits and/or disadvantages of care at home/inpatient care?

*Social influences/Environmental context and resources*

1. To what extent do/did you agree with your [patient] choice of rehabilitation setting after their surgery? (Prompt inpatient services and facilities, e.g., hospital bed, meals, home environment influence)

*Knowledge*

1. Do you know and can you describe what rehabilitation options (are/were) available to your [patient]?

*Beliefs about consequences*

1. What do you believe might happen to your [patient] if they were discharged directly home? [If preferred home or was discharged home - what do you believe will happen to them if they were discharged home earlier, such as day 3?]

*Emotion*

1. To what extent do/did emotional factors (e.g., worry, fear, anxiety) influence your preferred choice of rehabilitation for [patient].

*Beliefs about capabilities*

1. In what ways could the heath service make it easy/ier for [patient] or other patients to opt for care at home instead of inpatient care?

*Optimism*

1. How confident are you that care at home will result in a good outcome, just as good as inpatient care for your [patient]?

Part two: explore components of home-based services that may be acceptable to privately insured patients and their caregivers

1. To assist your [patient] and/or other patients to be discharged directly home instead of inpatient rehabilitation, what health care information would you need to know? How would this information be best delivered to you? When? By whom?
2. What health professional support might [patient] need at home? (Prompt for specific health care professionals, including interventions and intensity)
   - 1. Rehabilitation specialist or GP? If yes, what medical care would you need at home? (Prompt pain management). Any other specific medical reasons you need a doctor at home? How many visits per week? How many weeks?
     2. Nursing? If yes, what nursing care would you need at home? (Prompt pain or wound management), any other specific reasons you need a nurse at home? How many visits per week? How many weeks?
     3. Physiotherapy? If yes, what physiotherapy care would you need at home? (Prompt exercise therapy, exercises progressions) Any other specific reasons you need a physiotherapist at home? How many visits per week? How many weeks?
3. What other services or supports might [patient] need? (Prompt community services such as assistance with transport, shopping, meals, etc.)
4. Transport services? If yes, where to? (Prompt to/from hospital, grocery shopping, attend medical or physiotherapy appointments) What transport services (Prompt Taxi, Uber, other). How often? How many weeks?
5. Meals services? If yes, what type of meals? (Prompt meals on wheels, Uber eats) How many meals per day? How many weeks?
6. Cleaning service? If yes, what type of cleaning? How many services per week? How many weeks?
7. Self– care assistance? What type of self-care services? (Prompt Showering) How much self-care per day? How many weeks?
8. Is there any other services supports might [patient]/other patients need to be discharged directly home instead on inpatient rehabilitation?
