# Supplementary material for: Barriers, enablers and acceptability of home-based care following elective total knee or hip replacement at a private hospital: A qualitative study of patient and caregiver perspectives
Source: PLoS One. 2022 Aug 24;17(8):e0273405. doi: 10.1371/journal.pone.0273405 (PMC9401137; doi:10.1371/journal.pone.0273405)
Supplement: S3 Table — *Surgery cancelled (n = 1); #Proportion of patients for each variable per total number of patients; ^Proportion of patients receiving either inpatient rehabilitation or rehabilitation-at-home per number of patients for each variable. (DOCX) [file pone.0273405.s003.docx]

**S3 Table. Additional patient characteristics.**

| Variable | All patients (n=31*) | Received inpatient rehabilitation (n=13) | Received rehabilitation-at-home (n=17) |
| --- | --- | --- | --- |
| Body mass index, kg/m^2^ | Mean (SD) | Mean (SD) | Mean (SD) |
|  | 29.8 (5.4) | 30.9 (6.0) | 29.2 (5.0) |
| Insurance provider  Medibank Private  HCF  BUPA*  Australian Unity  GMHBA  CBHS Heath  La Trobe Health  AHM  HBF  Frank health  Defence health  Doctors Health Fund | N (%^#^) | N (%^^^) | N (%^^^) |
|  | 8 (26)  5 (16)  5 (16)  4 (13)  2 (6)  1 (3)  1 (3)  1 (3)  1 (3)  1 (3)  1 (3)  1 (3) | 3 (38)  4 (80)  2 (40)  2 (50)  0 (0)  1 (100)  0 (0)  1 (100)  0 (0)  0 (0)  0 (0)  0 (0) | 5 (63)  1 (20)  2 (40)  2 (50)  2 (100)  0 (0)  1 (100)  0 (0)  1 (100)  1 (100)  1 (100)  1 (100) |
| Comorbid conditions  Cardiac disease*  Previous cancer  Hypertension  Previous back surgery  Obstructive sleep apnea  Endocrine/thyroid condition  Persistent back pain  Respiratory condition  Gastrointestinal condition  Mental health condition  Central nervous system condition  Renal disease  Systemic arthritis  Lymphedema  Meniere’s disease | 8 (26)  6 (19)  4 (13)  4 (13)  3 (10)  3 (10)  2 (6)  2 (6)  2 (6)  2 (6)  2 (6)  1 (3)  1 (3)  1 (3)  1 (3) | 4 (50)  2 (33)  2 (50)  4 (100)  2 (67)  2 (67)  0 (0)  0 (0)  1 (50)  1 (50)  2 (100)  1 (100)  0 (0)  1 (100)  1 (100) | 3 (38)  4 (67)  2 (50)  0 (0)  1 (33)  1 (33)  2 (100)  2 (100)  1 (50)  1 (50)  0 (0)  0 (0)  1 (100)  0 (0)  0 (0) |
| Acute care complications if interviewed after surgery (n=16)  Any  Gastrointestinal  High temperature  Unstable blood pressure  Test for deep vein thrombosis  Dizziness episodes in standing  Blood transfusion | 9 (56)  2 (13)  1 (6)  3 (19)  3 (19)  1 (6)  1 (6) | 4 (44)  2 (100)  1 (100)  1 (33)  1 (33)  1 (100)  0 (0) | 5 (56)  0 (0)  0 (0)  2 (67)  2 (67)  0 (0)  1 (100) |

*Surgery cancelled (n=1); ^#^Proportion of patients for each variable per total number of patients; ^^^Proportion of patients receiving either inpatient rehabilitation or rehabilitation-at-home per number of patients for each variable.
