# Supplementary material for: Barriers, enablers and acceptability of home-based care following elective total knee or hip replacement at a private hospital: A qualitative study of patient and caregiver perspectives
Source: PLoS One. 2022 Aug 24;17(8):e0273405. doi: 10.1371/journal.pone.0273405 (PMC9401137; doi:10.1371/journal.pone.0273405)
Supplement: S4 Table — P—patient; C—caregiver; F—female; M—male; B—interviewed before surgery; A—interviewed after surgery. (DOCX) [file pone.0273405.s004.docx]

**S4 Table.** **Barriers and enablers of home-based care, with themes mapped to the Theoretical Domains Framework and illustrative quotes.**

| **Themes** | **Domains** | **Illustrative participant quotes** |
| --- | --- | --- |
| Feeling unsafe  Feeling confident | Emotion  Beliefs about capabilities  Beliefs about consequences | Patients received inpatient rehabilitation and their caregivers  “I live alone, and I’ve got stairs, and until I regain strength and mobility, I would be better off in rehab, than on my own” (P2, F, B)  “I was frightened to come home. It’s a big house and if you have to get up during the night to have to take the rubbish bins out, look after the dog poo, all the things that you have to do when you’re physically able to do it, and when I had the knee done, I’m not physically able to do it.” (P16, F, A)  “I would've found it very stressful to be at home that first week. I was in a lot of pain, very uncomfortable and I would've found it stressful as [caregiver] works as well” (P24, F, A)  “I am not afraid of the operation, I am just afraid that if I am not in the best place all the time, that something is going to go wrong.” (P13, F, B)  “My prime concern is the staircase and the fact that if she's in bed all the time when I go downstairs, if she wants to come downstairs and she's got the issue of moving on the staircase.” (C12, M, B)  “One of the things that I'd be really – and have been worrying to me, that if I was being relied on to give the drugs. I don't know anything about drugs. If I give two Panadol, fine, that's alright. but if I'm giving Endone or if I'm giving Palexia, I haven't gotten a clue what's that for” (C13, M, A)  Caregiver (patient cancelled surgery)  “When you exercise you do them incorrectly, you can do more damage than good if you’re not doing the exercise correctly” (C4, F, B) |
|  | Emotion  Beliefs about capabilities  Skills  Knowledge (procedural) | Patients received rehabilitation-at-home and their caregivers  “My husband actually is a medical practitioner so that may have relieved any anxiety I might have had if I've gone home without having that support. I had that confidence nothing is going to go really wrong here” (P27, F, B)  “I've got a good upper body strength and a good right leg to help me get through whatever I need to get through. I think I'm physically strong enough to be able to manage it” (P20, M, B)  “When we're completing the paperwork, he was 12 out of 12 on the – is it the RAPT score?” (C11, F, B)  “When you're in hospital, they had you on – a couple of days after the surgery, you’re on the stairs, so you were confident that you’re able to manage” (P29, F, A)  “I thought you had to be very careful what you did, and the physio said, ‘No, you’ve got to get out of bed and do your stuff’. So, that kind of information was incredibly useful” (C8, M, A)  “Really reaffirmed for me [Preadmission clinic] that I definitely want to come home” (P12, F, A) |
| Caregivers’ willingness to provide support | Social  influences | Caregiver (patient received rehabilitation-at-home)  “Whatever she needed, I would look after her but, I didn’t have a limit or an expectation” (C7, F, A)  Caregiver (patient received inpatient rehabilitation)  “If that’s her perception and she thinks that perception could be improved by going to rehabilitation, then that’s her choice. I’m happy for her to stay at home, but if she wants to go into rehab, I don’t have any problem” (C12, M, B) |
| Patients’ unwillingness to seek help | Beliefs about  consequences | Patients received inpatient rehabilitation  “You’re putting an enormous amount of pressure on the people around you who have to support you. I don’t want to punish the people around me” (P26, M, A)  “So, I’ve got children, but they’ve all got their own lives and I really don’t want to impinge on that” (P13, F, B)  “I don't think [husband] is going to be very happy about giving up some of the things he likes to do” (P22, F, B) |
| Less support and opportunity to rest | Environmental context and resources  Beliefs about  consequences | Patient received rehabilitation-at-home  “I did want to probably go into rehab only because I’ve got two teenage kids and if I come home, I would be more inclined to get up, get their lunch, get them out of – and I just wanted to rest” (P23, F, B)  Patients received inpatient rehabilitation  “They probably wouldn’t supervise you that much because they’d only be here for half an hour” (P16, F, A)  “You’re comparing a Holden to a BMW - you just can’t, they’re incomparable” (P26, M, A)  “The health care team that is available in rehabilitation is entirely the reason. The security of it, one feels safe and that you are doing whatever movements to the best possible ability to help the thing recover and those things wouldn’t happen if you weren’t somewhere where there are people there who are at the right time at the right place” (P7, F, B)    “I need a rest - I'd like to be not cooking and I just want to switch off” (P22, F, B)  “I wasn’t certain of because there’s a lot – certainly some confusion about what – so, if I’d come home, what services I would be able to get” (P21, F, A)  Caregiver (patient cancelled surgery)  “I just want him into rehab, so that they’ll monitor him a bit more closely and make sure that, you know, he's able to cope with exercises” (C4, F, B) |
| Positive feelings about home over the hospital | Emotion  Beliefs about consequences  Intentions | Patients received rehabilitation-at-home and their caregivers  “I’d much rather be at home in my own bed” (P15, F, B)  “Mum was going stir crazy after five [days in hospital]” (C7, F, A)  “I kept thinking in the first week, ‘What would I have done if I’d been there [inpatient rehabilitation]? I would have been in prison” (P31, F, A)  Patients received inpatient rehabilitation  “The only time I might change my mind about [rehabilitation-at-home] is if you told me corona virus, Ebola virus, was rampant through hospitals” (P26, M, A)  “I don't particularly like hospitals, so I'm not going there [inpatient rehabilitation] because I want to be in hospital” (P22, F, B) |
| Trusting specialists’ advice over family and friends | Social  influences | Patients received rehabilitation-at-home and caregiver  “Many people told me that I would be better off to have a rest after surgery and have people look after me, and that other people of my age and lifestyle found it very helpful. Well, I got a lecture from my sister-in-law that was convincing” (P31, F, A)  “Whilst people are very free with advice and well-meaning, that’s what happened historically and is useless information today” (P3, M, B)  “I'm still open to the fact that if I'm having difficulty post-surgery and they [Surgeon] suggest that I go to rehab – “Oh, he'll go” (C11, F, B) – “Then I’ll go” (P20, M, B)  Patients received inpatient rehabilitation  “If my specialist said that that would be okay for me, then yes, I possibly would be interested” (P25, F, B) |
| Certainty about anticipated recovery | Knowledge  Optimism  Beliefs about consequences  Beliefs about capabilities | Patients received rehabilitation-at-home  “I have the advantage that I’ve been through it once” (P15, F, B)  “I thought by having physio come into my home they would arrive at a set time, rather than being in a group and not having that one-on-one attention” (P11, F, A)  “I really want to be on my feet and doing what I should do in as short a time as possible” (P10, F,A)  Patients received inpatient rehabilitation  “The outcome’s unknown at the moment for me. I don’t know what he will do, how my leg might be. Everything might go haywire” (P25, F, B)  “I don’t know what exactly it feels like after two or three days or four days in hospital, whether because I’ve sort of built myself up in strength – physical strength to be able to cope with recovery afterwards, whether I might be well enough to cope with being by myself. It’s a question which I can’t answer now” (P4, M, B) |
| Length of hospital stay | Environmental context and resources | Patients received rehabilitation-at-home  “That extra couple of days [in acute hospital] helps to get the drugs out of the system and the anaesthetic and everything else like that and you could walk through or walk around the hospital a few times, and get extra physio there because you had people there on the weekend” (P17, M, A)  “When I learnt that I would have had to stay there [inpatient rehabilitation] seven days or ten and I didn’t want to do that. I would have been happy to go for three [days], but not for seven or ten.” (P31, F, A) |
| Paying for health insurance | Environmental context and resources | Patient received inpatient rehabilitation and caregiver  “I pay for all this insurance, I’m going to make the most of it” (P26, M, A)    “He has private insurance and all covered. Why to send him home?” (C3, F, B)  Patient received rehabilitation-at-home and caregiver  “If people went straight home, they either had to visit a physio or they would have a physio come once a week to home depending on what their insurance covered - so that didn’t impress me at all’ (P5, F, B)  “Even if it's included in the health cover, it's not an expense we need to make anyone incur because he is younger than most people who have a hip replacement done and he is healthier than majority of the people who are gonna have it done” (C11, F, B)    “We’re in a position where we’re fortunate where we can pay [for home-based care] now” (C5, F, B) |

P – patient; C – caregiver; F – female; M – male; B – interviewed before surgery; A – interviewed after surgery
