# Supplementary material for: Barriers, enablers and acceptability of home-based care following elective total knee or hip replacement at a private hospital: A qualitative study of patient and caregiver perspectives
Source: PLoS One. 2022 Aug 24;17(8):e0273405. doi: 10.1371/journal.pone.0273405 (PMC9401137; doi:10.1371/journal.pone.0273405)
Supplement: Attachment — Submitted filename: To the editor.docx [file pone.0273405.s006.docx]

To the editor

Thank you for giving me the opportunity to review this manuscript which is very interesting and covers an interesting subject.

The aim of the study is valuable and important for the health system and providers designing home programs after TKR and THR.

I have a few questions and suggestions for changes.

**Abstract**

Methods

Please include the total number of semi-structured interviews

Please describe the participants characteristics e.g. mean age (SD), and % sex for both patients and caregivers

Conclusion

Typo: ”TKR or TKR” – one should be THR

**Introduction**

Please state that the majority of joint replacements in Australia are performed in the private sector, as it can be different in other countries.

**Methods**

Sampling

Please, can you elaborate the participant recruitment. How did you select the participants at the pre-admission information session– were they consecutively selected during the study period? Did you approach them one-by-one, or as a group?

Setting

Where did the interviews take place? Location, setting and was it a face-to-face interview? And was anyone else present during the interview?

Interview guide

How did you conduct the interview guide? (how did you come up with the questions) And did you adjust the research questions as you went along with the interviews? Or did you use the same questions for all interviews? And was the interview guide pilot tested prior to study commencement?

Furthermore, were field notes made during/after interview?

Researcher characteristic

Please elaborate the personal characteristics of the interviewer. Clinical background/occupation, credentials, sex, years of experience within this field of research? Experience with qualitative research?

What was the participants knowledge of the interviewer? Did they know about the researcher? E.g. personal goals, reason for doing the study? The purpose of the study?

**Results**

In the text it is stated: “one patient interviewed prior to a planned THR cancelled surgery”. However, in Table 1 it looks like it is a patient with TKR that is missing. See table 2, Under “interviewed before surgery/surgery type”. Please elaborate or modify accordingly.

Before describing the results, please sum up the number of barrier and enabler themes found, and the number of acceptability themes.

Please, provide more information in the text on the participants when describing barrier and enabler themes. In particular, I would like you to add after each quote whether the participant is a patient or caregiver, a female or a male, interviewed before or after surgery, RAH or inpatient – just like you have done in table 2. This is especially relevant if the quote is not listed in Table 2.

I´m a bit confused of how and when you use the quotation in the text under the barrier and enabler themes. Under the headline “Feeling unsafe versus feeling confident” I would like at least, one quote in the text that verifies that patients who received RAH felt safe – I do not find Table 2 sufficient here.

For the two following headlines the quotations in table 2 supports your text. Then, under the headline “Positive feelings about home over the hospital” you suddenly use the same quotation both in the text and in table 2.

Please align how and when you use the quotations in the text

Line 290: Typo (delete “patients and”) it is stated twice.

**Discussion**

Typo in the second line in the discussion section, “TKR or TKR” one should be THR.

What does the abbreviation “THA” and “TKA” stand for?

In the discussion you state that there are many similar findings from other studies across the world. Please, elaborate the similarities/strengths to this study, and discuss the weaknesses in relation to other studies, discuss particularly any differences in results.

Please, do also discuss your findings regarding who chose to receive rehabilitation at home vs. inpatient rehabilitation (table 1).

The participants who chose rehabilitation at home were younger, still employed, lived with a caregiver and had a higher RAPT-score. What does this mean, and what implication does it have for your clinical practice.

Does the study leave any unanswered questions, and/or do you have any thoughts about future research questions/topics?

**Conclusion**

I question whether you have the data to conclude that it is a patient behavioral change strategy that is needed to enable an increased uptake and acceptability of HAM and RAM.

In the discussion section, you sum up that it is components of care (pre-discharged home visits, information on recovery, access to health care support to ensure safe mobility and efficient exercise at home, and tailored domestic support) that is needed.

The solution of a behavioral change strategy was introduced as a suggestion in the discussion section. Please elaborate or modify accordingly.
