# Supplementary material for: Barriers, enablers and acceptability of home-based care following elective total knee or hip replacement at a private hospital: A qualitative study of patient and caregiver perspectives
Source: PLoS One. 2022 Aug 24;17(8):e0273405. doi: 10.1371/journal.pone.0273405 (PMC9401137; doi:10.1371/journal.pone.0273405)
Supplement: Attachment — Submitted filename: Response to Reviewers (1).docx [file pone.0273405.s007.docx]

Carsten Bogh Juhl, PhD

Academic Editor

PLOS ONE

Re:	PONE-D-21-32322

Barriers, enablers and acceptability of hospital-at-home and rehabilitation-at-home following elective total knee or hip replacement at a private hospital: A qualitative study of patient and caregiver perspectives

**Additional editor comments**

The reviewers have found your study interesting - but have some important suggestions - especially clarifying the method and especially align the conclusion with the available data as these only represent a selected group of patients.

**Author response:** Thank you for the opportunity to submit a revised version of the manuscript. We greatly appreciate the reviewers’ comments and consider addressing these have led to an improved manuscript. We can confirm the manuscript meets PLOS ONE's style requirements, including those for file naming. Please find our response to the reviewers’ comments below. We have also included the manuscript changes and used red type below to highlight the relevant changes to the manuscript text.

**Reviewer #1**

This paper identified barriers and enablers of hospital-at-home and rehabilitation-at-home in privately insured patients receiving total knee and hip replacement. Furthermore, components of care that may increase the acceptability of hospital-at-home and rehabilitation-at-home were identified.

Overall, this study provides an interesting insight into barriers, enablers and acceptability of hospital-at-home and rehabilitation-at-home. The authors used interviews for exploring privately insured patients’ and caregivers’ perspectives and used triangulation in both the data collection and in the analysis. The authors presented the findings in a thick description and related the findings to findings in other studies in the discussion.

However, some concerns may be raised related primarily to the introduction and the method section. These concerns must be addressed to increase the credibility of this study.

**Reviewer #1:** The authors mentioned that hospital-at-home and rehabilitation-at-home facilitates earlier transition to home and provides similar results compared to inpatient care. Furthermore, the demand for inpatient rehabilitation in the private sector will increase, but it is mentioned in the discussion, that it is unknown whether the administrators of private hospitals see hospital-at-home and rehabilitation-at-home as beneficial. Therefore, the reason for carrying out this qualitative study in a private setting can be mentioned more explicitly.

**Author response:** In the revised introduction, we have been more explicit about the reason for conducting this qualitative study in a private setting in Australia.

We agree it is important to understand staff, including administrator, perspectives on whether they consider these models to be beneficial, and this is a topic for future research. At our private hospital, the orthopaedic surgeons share the view that patient and caregiver preferences are key drivers of inpatient rehabilitation. Also, staff and administrators see the benefits of offering home-care options to patients, including freeing up inpatient bed capacity. A previous qualitative study conducted in an Australian private hospital setting has also identified consumer preferences as a strong driver of inpatient rehabilitation. Reference to this study is included in our revised manuscript. This previous study, however, didn’t use a theoretical approach to explore barriers and enablers to home-based care, and by doing so may have overlooked potentially important factors influencing change needed for designing tailored strategies to increase uptake in the private setting.

**Author change:** ‘One previous qualitative study exploring clinician and patient decision-making for rehabilitation following THR and TKR in private hospital settings in Australia identified patient preference for inpatient rehabilitation as a key barrier to home discharge (17). However, this study did not use a theoretical approach to explore barriers & enablers to home-based care in the private setting. Using a theoretical framework facilitates a more thorough assessment of the factors influencing implementation and can assist in designing tailored strategies likely to achieve greater uptake of hospital-at-home and rehabilitation-at-home (18-20).’ (Page 5, Lines 97-104)

**Reviewer #1:** The authors used the Consolidated criteria for reporting qualitative studies (COREQ) checklist for their reporting (line 101-102) which is a relevant checklist for qualitative studies. Several points related to the research team and reflexivity are not fully described in the paper.

**Author response:** We have thoroughly checked the manuscript in relation to each item from Domain 1 of the COREQ checklist (Research team and reflexivity), and have revised the manuscript to ensure all of the items are fully described. In this resubmission, we have also attached the COREQ checklist as a Supplementary File (S1 File).

**Author change:** ‘All interviews were conducted by one male author (JW), an experienced physiotherapist (20 years) providing care at the hospital, who has in-depth knowledge of the hospital services and is a postdoctoral research fellow with experience in qualitative research. None of the participants were known to the interviewer.’ (Pages 9-10, Lines 208-11)

**Author change: ‘**All authors have knowledge that hospital-at-home and rehabilitation-at-home interventions are underutilised, yet with similar effectiveness compared to inpatient care.’ (Page 11, Lines 241-3)

**Reviewer #1:** The authors used interviews for exploring patients’ and caregivers’ perspectives. On this basis, I assume, that a phenomenological approach was taken, however, the approach is not described in the paper. Please identify the approach adopted to this qualitative study.

**Author response:** Our methods involved a descriptive qualitative approach and semi-structured questions aimed at exploring participants’ views on home-based care. We have added this approach to the design section of the methods with the following text and citations:

**Author change:** ‘A qualitative descriptive study involving semi-structured interview questions aimed at exploring participants’ perspectives on home-based care was conducted. (23, 24)’ (Page 6, Lines 118-9)

**Reviewer #1:** The authors obtained study approval and the informants gave signed informed consent prior to commencement of the study. It is recommended to add this information to the manuscript.

**Author response:** We have added this to the text as requested.

**Author change:** ‘Ethics approval was obtained from the Cabrini Health Human Research Ethics Committee prior to commencement of the study (02-28-10-19). All participants provided written, informed consent prior to their participation in the interview.’ (Page 6, Lines 119-22)

**Reviewer #1:** In the selection process of informants purposive sampling was used (line 123-124) – a relevant approach for achieving an insight into the phenomenon of interest. The objective is to explore barriers and enablers of both rehabilitation-at-home and hospital-at-home (line 92-95), but only the selection criteria: inpatient rehabilitation and rehabilitation-at-home were used (line 123-124). As reported in the results none of the informants received hospital-at-home, thus the perspective from patients receiving hospital-at-home and their caregivers are missing.

**Author response:** We purposively sampled patients who identified as preferring rehabilitation-at-home or inpatient care based on patient’s response to the open-ended question on the Risk Assessment and Prioritisation Tool (RAPT). We have clarified this in the recruitment section of the revised manuscript.

The interview guide contained questions exploring home-based services rather than specific hospital-at-home and rehabilitation-at-home models. Given that we did not explicitly differentiate between hospital-at home and rehabilitation-at-home, we have amended the objective of the study to exploring home-based care.

Furthermore, at the time of participant recruitment, the hospital-at-home service was not currently utilised for this patient cohort. As the perspective from patients having received hospital-at-home and their caregivers are missing, we have clarified this in the recruitment section of the methods and added as a limitation in the discussion.

**Author change:** ‘To ensure a range of views were explored in this study, purposive sampling was used to include patients with their preferred discharge destination as either inpatient or home using the RAPT.’ (Page 8, Lines 180-2)

**Author change:** (i) explore barriers and enablers to implementation of home-based care… (ii) explore components of home-based care…(Page 5, Lines 107-9)

**Author change: ‘**At the time of study recruitment, hospital-at-home was not utilised for this patient cohort and provides an alternative option to acute inpatient care’ (Page 7, Lines 139-41)

**Author change:** ‘The interview guide did not explicitly differentiate between hospital-at home and rehabilitation-at-home, the perspective from patients having received hospital-at-home and their caregivers are missing...’ (Page 28, Lines 636-8)

**Reviewer #1:** The interview guide was guided by the Theoretical Domains Framework (line 129-131). Using a framework facilitates a thorough assessment of a phenomenon of interest in this case barriers and enablers of hospital-at-home and rehabilitation-at-home. Not all domains of the Theoretical Domains Framework were covered by questions in the interview guide (Table S1, S2) and a reason for this is not described in paper. Furthermore, it is recommended and usual practice to pilot test the interview guide. It is left unclear whether the interview guide has been pilot tested in this study (line 129-138). It would be useful to know have more information about this.

**Author response:** We carefully considered the balance between including questions on all the domains versus focusing on the domains that appeared most relevant to the barriers and enablers of uptake of home-based care. We chose to adopt the latter approach, as recommended by Atkins et al., 2017 ‘A guide to using the Theoretical Domains Framework of behaviour change to investigate implementation problems.’ To minimise the chances of missing any potentially relevant factors, we also included a number of ‘catch all’ questions that sought to elicit data about any domain (e.g., ‘are there any other factors that influenced your preference for rehabilitation’ and ‘are there any other comments you’d like to make about rehabilitation-at-home or in the hospital?’). As we did not include questions for every domain in the interview guide, only those considered most relevant, we cannot exclude the possibility that factors associated with these domains don’t exist. Therefore we have added this as a limitation in the discussion.

The interview guide was not pilot tested with patients or caregivers prior to data collection and this has also been added as a limitation. The interview guide was developed by our multidisciplinary team with clinical expertise in joint replacement surgery and rehabilitation, as well as academic expertise in qualitative and implementation research to ensure it was clinically sensible and comprehensible. We have clarified these data collection methods in the manuscript.

**Author change: ‘**We explored the theoretical domains considered most relevant to the uptake of home-based care’ (18) (S1and S2 Tables). (Page 9, Lines 190-1)

**Author change: ‘**The interview guide was developed by our multidisciplinary team with clinical expertise in joint replacement surgery and rehabilitation, as well as academic expertise in qualitative and implementation research to ensure it was clinically relevant and comprehensible. The same interview questions were used for all interviews, and field notes were taken during and after interviews. The interview guide was not pilot tested prior to data collection.’ (Page 9, Lines 196-201)

**Author change: ‘**The interview guide … was not pilot tested.’ (Pages 28, Lines 638)

**Author change: ‘**As we did not include questions for every TDF domain in the interview guide, only those considered most relevant, we cannot exclude the possibility that factors associated with these domains do not exist.’ (Page 28-9, Lines 639-41)

**Reviewer #1:** The patients were invited to participate in the study at the preadmission information session (line 122-123), but in what way they were invited and how many patients refused to participate is unclear.

**Author response:** The pre-admission session presenter informed attending patients and caregivers about the study verbally and provided them with a study flyer in their information packs. If any patients and caregivers were interested in participating, they were invited to provide their contact details on the flyer or contact the primary investigator (JW) who was available at the end of each session or via phone. We have clarified this method in the revised manuscript.

We have also calculated the response rate based on the number of patients attending the sessions during the recruitment period. As we do not know if all patients who attended the sessions were eligible, the response rate is likely an underestimate and has been added as a limitation. Also the caregiver response rate could not be calculated as the number attending the sessions was not recorded. This has also been added as a limitation.

**Author change:** **‘**We invited participation in the study at consecutive group-based, face-to-face preadmission information sessions, conducted weekly at the hospital. A hospital-employed clinician presenter informed attending patients and caregivers about the study verbally, and provided a study flyer in patients’ information packs. If any patients and caregivers were interested in participating, they returned an expression of interest or contacted the primary investigator (JW) available at the end of each session or via phone.’ (Pages 7-8, Lines 159-64)

**Author change:** The patient response rate to participate was 26%. All patients who expressed interest participated in the interviews, except for one female patient who was ineligible due to having a partial knee replacement. (Page 11, Lines 248-50)

**Author change:** The response rate for patient participation may be underestimated as we did not know if all patients attending the preadmission sessions were eligible, and the caregiver response rate was not calculated. (Page 28, Lines 633-6)

**Reviewer #1:** The interviews were undertaken between November 2019 and March 2020 (line 182), but please describe the setting in which the interviews took place.

**Author response:** The interviews were conducted in the patient’s home (19 interviews), or via telephone (20 interviews) depending on the participants’ preferences and were conducted either one-to-one or jointly with the patient and their caregiver.

**Author change:** ‘Interviews were conducted face-to-face in participants homes or via the telephone, depending on participants preferences. Interviews were conducted one-to-one or in dyads (i.e., patient with their caregiver) depending on participant preferences.’ (Page 9, Lines 203-5)

**Author change:** ‘Data saturation was achieved after 39 interviews (20 face-to-face, 19 via telephone)’ (Page 11, Lines 246-7)

**Reviewer #1:** The discussion summarizes the finding and mention influential theoretical domains for the barriers (discussion, line 5-7). It is also suggested to mention the influential theoretical domains after summarizing the enablers (discussion, line 7-10).

**Author response:** The key domains for the enablers have been summarised in the discussion in the revised manuscript.

**Author change:** ‘Theoretical domains relating to these enablers included ‘beliefs about capabilities’, ‘skills’, ‘procedural knowledge’ and ‘social influences.’ (Page 25, Lines 549-50)

**Reviewer #1:** In the fourth section of the discussion the authors wrote: “Patients and caregivers in our study had a low desire for medical and domestic support at home and a greater desire for home physiotherapy….”. The last four quotes in Table 3 indicates a desire for domestic support at home under the theme “No one size fits all model for domestic support” to increase the acceptance of hospital-at-home and rehabilitation-at-home indicates a bit of a contrast. It is suggested to rephrase.

**Author response:** Thank you for identifying this. We have rephrased the sentence in the revised manuscript.

**Author change:** ‘Patients and caregivers in our study had a high desire for home physiotherapy including information about recovery at home. Domestic support was desired for patients without caregiver support, and medical support was deemed needed in the case of complications.’ (Page 26, Lines 581-3)

**Reviewer #1:** The authors aimed at exploring the perspectives of privately insured patients at a private hospital and only privately insured patients participated in this study. It is recommended to mention in the conclusion (page 29) that these are privately insured patients.

**Author response:** We agree and have amended the manuscript accordingly.

**Author change:** ‘Multiple factors emerged that influence implementation of home-based care from the perspectives of privately insured patients and caregivers …’ (Page 29, Line 651)

**Reviewer #1:** **Some minor suggestions for the tables:**

**Reviewer #1:** Table 1 – Caption: please chose either patient or participant.

**Author response:** We have selected patient and revised the caption for Table 1 accordingly.

**Reviewer #1:** Table 1 – Surgery type: 20 patients having total knee replacement were interviewed. Nine patients received inpatient rehabilitation and 10 patients received rehabilitation-at-home (= 19 patients). I presume, that the * should be placed at TKR and not at “Interviewed before surgery”.

**Author response:** We can confirm that the patient who cancelled surgery was scheduled for a total knee replacement and was also interviewed before surgery (i.e., 15 patients were interviewed before surgery, 8 patients received inpatient rehabilitation and 6 patients received rehabilitation-at-home = 14 patients). Therefore, we have added the * after TKR, and can confirm the * can remain at “interviewed before surgery”

**Author change:** TKR* (Page 13)

**Reviewer #1:** Table 1 – Please provide an explanation for the abbreviations TKR and THR.

**Author response:** TKR (total knee replacement) and THR (total hip replacement) has been added to Table 1.

**Author change:** TKR = total knee replacement; THR= total hip replacement (Page 14, Lines 275-6)

**Reviewer #1:** Table 2 – I have a bit of a hard time seeing which quotes belong to which theoretical domain in a few sections for example in the section “Feeling unsafe” - can it be made more manageable?

**Author response:** We have amended Table 2 to better align the domains with the quotes. Based on the reviewers’ feedback, Table 2 and 3 have now been included as Supplementary Tables.

**Author change:** Please refer to S4 and S5 Tables.

**Reviewer #1:** Table 2 – The first section includes quotes from patient receiving inpatient rehabilitation. But in what way can the patient who cancelled surgery (last quote) be an inpatient? Suggest doing it as the authors did in the section: “Less support and opportunities to rest” (last quote).

**Author response:** We have amended the Table as suggested.

**Author change:** Please refer to S4 Table.

**Reviewer #2**

Thanks for the opportunity to read your article and make a review. This article addresses a topical issue in terms of patients and caregivers’ perspectives on rehabilitation-at-home and provides information about barriers and enablers for this intervention. It is important to consider participants’ views on the intervention, as acceptability may undermine the uptake and thus the implementation. Strengths of this study includes the qualitative approach that is underutilized, the inclusion of both patients and caregivers as well as use of TDF.

The manuscript is well written and the presentation comprehensive and detailed, but there are some points that should be clarified.

**Reviewer #2:** The authors should clarify definitions and differences between the different interventions and concepts around which the article circulates. This will help to provide an understanding of the authors’ preconceptions as well as clarify the difference between a statement classified as an enabler and a statement classified as a theme about an acceptable component.

**Author response:** In the revised methods, we have clarified the definitions and process of classifying themes as barriers/enablers, and as acceptable components of care. In the introduction we have also now included the definitions of implementation and acceptability used in this study.

**Author change:** ‘In this study we used the term implementation to describe the uptake of best care, evidence-based practices into routine practice with the aim of improving patient care (21). We used the term acceptability to reflect the extent to which people receiving a healthcare intervention consider it to be appropriate, based on anticipated or experienced cognitive and emotional responses to the intervention (22).’ (Pages 5-6, Lines 110-4)

**Author change:** ‘We used the concept of ‘barriers and enablers to implementation' as the themes related to patients’ uptake of home-based care’ (Pages 10-11, Lines 235-7)

**Author change:** ‘For acceptability, these themes related to how patients and caregivers considered the components of home-based care to be appropriate, based on anticipated or experienced cognitive and emotional responses to the intervention.’ (Page 11, Lines 238-41)

**Reviewer #2:** In the introduction inpatient rehabilitation is described, but rehabilitation-at-home and hospital-at-home is not elaborated to the same extent although the components are central to the study. Knowing the components of the interventions and the extent to which patients and caregivers’ have knowledge of the interventions and their content, is important to assess the interviewees statements and for assessing the relevance to their own practice for the international reader. The components of the interventions may be described briefly in the section about setting and be detailed in an additional file.

**Author response:** In our study, the interview guide contained questions exploring home-based services rather than specific hospital-at-home and rehabilitation-at-home models. To clarify the hospital services we have provided more details of these interventions in the methods section (Setting) of the revised manuscript. Alternatively, if the editor prefers, we could provide these details in the Supplementary Files.

**Author change:** ‘Following surgery, acute inpatient care was typically provided for up to five days and then patients were referred to either rehabilitation-at-home, outpatient rehabilitation or inpatient rehabilitation. Within the multiple payer system, patients may be eligible for some or all services depending on their insurance coverage. At the time of study recruitment, hospital-at-home was not utilised for this patient cohort and provides an alternative option to acute inpatient care. For hospital-at-home, patients remain admitted as acute patients where they have a short acute inpatient stay (2 days) followed by daily care from a physician, nurse, and physiotherapist (all employed by the hospital) in their homes for a limited time (typically 2 to 3 days). These patients are then referred onto rehabilitation-at-home or outpatient physiotherapy following discharge from hospital-at-home’ (Page 7, Lines 136-45)

‘For rehabilitation-at-home, patients are discharged from acute care to the hospital service (‘Therapy in the Home’ with staff employed by the hospital) or an external private home rehabilitation service. Rehabilitation-at-home programs are provided for about 4 to 6 weeks by allied health professionals and include negotiated goals aiming to optimise the patient’s functioning and quality of life, caregiver support and education. For outpatient rehabilitation, patients may attend a facility such as a private physiotherapy practice (may include home visits) or an outpatient rehabilitation service. For inpatient rehabilitation, patients receive subacute care in the dedicated rehabilitation ward or an external hospital, and length of stay is typically about 10 days. Following inpatient rehabilitation, patients are commonly referred to outpatient rehabilitation at either the hospital outpatient facility or to an external facility’ (Page 7, Lines 147-56)

**Reviewer #2:** The authors should elaborate and/or discuss hospital-at-home, as it is unclear whether hospital-at-home is studied, since the intervention is sparsely described, the interview guides (S1 and 2) use the term “home-based care”, it is unclear if the patients have knowledge about the intervention and no patients receive hospital-at-home (which is reflected in tables 1 and S3 and furthermore the purposive sampling doesn’t include hospital-at-home participants (p.6, l 122-124)). The authors may consider adding and discuss information on whether hospital-at-home and rehabilitation-at-home are interventions that are offered to all patients undergoing surgery in the private / public sector in Australia (or the private hospital where the study takes place).

**Author response:** The interview guide contained questions exploring home-based services rather than specific hospital-at-home and rehabilitation-at-home models. Given that we did not explicitly differentiate between hospital-at home and rehabilitation-at-home, we have amended the objective of the study to exploring home-based care. As hospital-at-home was not utilised during the study period, patients and caregivers were unaware of this service. We have clarified this in the recruitment section of the methods and added as a limitation in the discussion. We have also added additional information to describe hospital-at-home and rehabilitation-at-home to give readers a sense of the relevant services at the private hospital where our study took place.

**Author change:** (i) explore barriers and enablers to implementation of home-based care … (ii) explore components of home-based care… (Page 5, Lines 107-9)

**Author change: ‘**At the time of study recruitment, hospital-at-home was not utilised for this patient cohort…’ (Page 7, Lines 139-40)

**Author change:** ‘The interview guide did not explicitly differentiate between hospital-at home and rehabilitation-at-home, the perspective from patients having received hospital-at-home and their caregivers are missing...’ (Page 28, Lines 636-8)

**Author change: ‘**Following surgery, acute inpatient care was typically provided for up to five days and then patients were referred to either rehabilitation-at-home, outpatient rehabilitation or inpatient rehabilitation. Within the multiple payer system, patients may be eligible for some or all services depending on their insurance coverage. At the time of study recruitment, hospital-at-home was not utilised for this patient cohort and provides an alternative option to acute inpatient care. For hospital-at-home, patients remain admitted as acute patients where they have a short acute inpatient stay (2 days) followed by daily care from a physician, nurse, and physiotherapist (all employed by the hospital) in their homes for a limited time (typically 2 to 3 days). These patients are then referred onto rehabilitation-at-home or outpatient physiotherapy following discharge from hospital-at-home’ (Page 7, Lines 136-45)

**Reviewer #2:** The second aim of the study concerns patients’ experience of components influencing the acceptability of the rehabilitation-at-home and hospital-at-home. The authors may consider to accurately describe acceptability and components influencing acceptability – what is the rationale for selection of the specific components (e.g., information and support by health professionals that are components asked about in the interview guide) and is it theoretical underpinned (data collection p. 7, l 133-138)?

**Author response:** Acceptability related to how patients and caregivers considered the components of the home-based care to be appropriate, based on anticipated or experienced cognitive and emotional responses to the intervention. There was no theoretical underpinning for this part of the study. We asked about information and professional support, but also included an open-ended question about ‘any other services or supports that may be needed.’ The intervention components selected for the interview guide were developed by our multidisciplinary research team.

**Author change:** ‘For acceptability, these themes related to how patients and caregivers considered the components of home-based care to be appropriate, based on anticipated or experienced cognitive and emotional responses to the intervention.’ (Page 11, Lines 238-41)

**Author change:** ‘The interview guide was developed by our multidisciplinary team with clinical expertise in joint replacement surgery and rehabilitation, as well as academic expertise in qualitative and implementation research to ensure it was clinically relevant and comprehensible…’ (Page 9, Lines 196-9)

**Reviewer #2:** Implementation is used inconsistently in literature and across disciplines, thus an elaboration in this study is requested especially as implementation is a part of the conclusion. It is uncertain whether implementation is seen primarily as uptake to the interventions, as this is the primary argument for the purpose of the study. Does it e.g., primarily focus on the mechanism through which delivery is achieved.

**Author response:** We used the concept of implementation to describe the uptake of best care and other evidence-based practices into routine practice with the aim of improving patient care (Grimshaw 2013).

**Author change:** ‘In this study we used the term implementation to describe the uptake of best care, evidence-based practices into routine practice with the aim of improving patient care (21)’ (Pages 5-6, Lines 110-2)

**Reviewer #2:** The authors should clarify why the use of data from RAPT is important in this qualitative study – do the authors expect it to have an impact on patients’ and/or caregivers’ statements or the interpretation of these? The authors might include a discussion if the RAPT results were as expected and whether it has had an impact on the statements / interpretations (P. 7, l. 147- and p. 9, l. 193-).

**Author response:** In our study, 11 patients with a lower RAPT score of 6 to 9 did not receive rehabilitation-at-home as predicted by their score. Instead they had received inpatient rehabilitation. Common barriers to rehabilitation-at-home in this cohort included feeling unsafe, uncertainty about recovery and preferring to rest, and therefore impacted on the uptake of home-based care. These findings have implications for future research and this has been included in the discussion.

**Author change: ‘**They could be targeted to specific patients who prefer inpatient rehabilitation, as indicated by a lower RAPT score (i.e., 6 to 9), those living without a caregiver and retired.’ (Page 28, Lines 621-2)

**Reviewer #2:** Title and conclusion: The title and conclusion are appropriate if the above comments on acceptability and hospital-at-home can be substantiated in the article.

**Author response:** We believe we have carefully addressed the comments on acceptability and hospital-at-home. As the interview guide did not explicitly differentiate between hospital-at home and rehabilitation-at-home, we have amended the title and conclusion accordingly.

**Author change:** ‘Barriers, enablers and acceptability of home-based care following elective total knee or hip replacement at a private hospital: A qualitative study of patient and caregiver perspectives. (Title)

**Author change:** Multiple factors emerged that influence implementation of home-based care from the perspectives of privately insured patients and caregivers that give valuable insight for health systems and providers designing suitable home programs.’ (Conclusion, Page 29, Lines 650-52)

**Reviewer #2: Minor details to consider:**

**Reviewer #2:** P. 6, l. 107-: Consider using the term caregivers to clarify the transition from patient to caregiver

**Author change:** Adult caregivers who would primarily help with activities of daily living… (Page 6, Lines 128-9)

**Reviewer #2:** P. 8, l.155-166: Missing source at last line.

**Author response:** Reference 27 (Sconza 2019) was added as the source**.**

**Author change:** ‘Lower scores (< 6) are indicative of higher risk for inpatient rehabilitation (27)’ (Page 8, Line 179-80)

**Reviewer #2:** P. 8, l. 158: Body mass index is part of Table 1 and not mentioned in results or discussion – consider relevance or moving to S3.

**Author response/change:** We have moved this characteristic to S3 Table.

**Reviewer #2:** P. 8, l. 175: “All authors” – is it a reference to all 9 authors?

**Author response:** Yes, this was all 9 authors in a meeting.

**Author change:** ‘All nine authors discussed the emergent themes…’ (Page 10, Line 233)

**Reviewer #2:** P. 14, table 2, first quote under the heading “Intentions”: The patient uses the term “the only time I might change my mind about RAH is if you told me corona virus…” – are those the words that support the result that “the hospital was a place where there was a risk of catching the contagious virus”?

**Author response:** Yes, we can confirm this. This quote has now been included in the text. (Page 18, Lines 378-80)

**Reviewer #2:** P. 25: In the first lines of the discussion “TKA or TKA” probably should be corrected to “THA and TKA”

**Author response:** We have now corrected these typos to THR and TKR.

**Author change:** ‘as perceived by privately insured THR and TKR patients.’ (Page 24, Lines 543-4)

**Reviewer #3**

A language revision is recommended throughout the manuscript. Please check grammar and spelling - there are several typos in the manuscript. E.g., a typo in the second line in the discussion section, “TKR or TKR” one should be THR.

**Author response/change:** We have re-read and checked the grammar and spelling. We have corrected the typos for TKR in two places. We have replaced the term carer with caregivers for consistency. We have deleted words considered redundant, such as ‘particular’, and not abbreviated hospital-at-home and rehabilitation-at-home.

**Reviewer #4**

Comments to the authors: Overall, the manuscript is well-written with detailed information about methodological approach and considerations. Please see the comment below regarding each manuscript section.

**Abstract**

**Reviewer #4:** The abstract clearly states the rationale for conducting the study, the aim, results and conclusion in a precise way that inclines me to further reading.

**Introduction**

**Reviewer #4:** The introduction gives a clear description of the patient population, health care setting and potential benefits of at-home rehabilitation.

**Reviewer #4:** The aim of the study is clear, however, it would be informative to include the authors hypothesis, as this will strengthen the transferability of the study.

**Author response**: We were not testing a hypothesis in this qualitative interview study, but rather we sought to explore and describe the factors influencing uptake of home-based care from the perspectives of patients and caregivers.

**Methods**

**Reviewer #4:** The sampling strategy seems appropriate according to the study aim.

**Reviewer #4:** Data collection; there is a need for additional information regarding data collection, including the interview setting, i.e. face-to-face, hospital setting, at home, telephone? Was the setting the same for all participants, or was is based on the participants’ preferences?

**Author response:** We have included the additional information regarding data collection in the revised manuscript.

**Author change:** ‘Interviews were conducted face-to-face in participants homes or via the telephone, depending on participants preferences. Interviews were conducted one-to-one or in dyads (i.e., patient with their caregiver) depending on participant preferences.’ (Page 9, Lines 203-5)

**Reviewer #4:** It was nice to read that validations of transcripts were made by the participants.

**Reviewer #4:** The interviewer’s role related to the participants was not stated. Was the interviewer known to the participants before the interview? This is crucial due to risk of bias, pre-understanding, and influence during the research process. Should also be included in the discussion section.

**Author response:** The interviewer was an experienced physiotherapist who worked at the private hospital, however none of the participants were known to the interviewer. We have included these details in the revised manuscript.

**Author change:** ‘All interviews were conducted by one male author (JW), an experienced physiotherapist (20 years) providing care at the hospital, who has in-depth knowledge of the hospital services and is a postdoctoral research fellow with experience in qualitative research. None of the participants were known to the interviewer. (Pages 9-10, Lines 208-11)

**Reviewer #4:** Line 129: It is stated that the interviews are semi-structured. The supplementary material show that the interview guide is quite comprehensive and includes very specific questions. Suggest describing the interview procedure more in detail related to use of the interview guide, and/or referring to the interviews as “structured”?

**Author response:** We have clarified that the interviews were semi-structured as the as the order of questions in the interview guide was flexible and adapted by the interviewer to fit the flow of conversation with participants.

**Author change:** The semi-structured interview guided was informed by the Theoretical Domains Framework (TDF). The order of questions in the interview guide was flexible and adapted by the interviewer (e.g., ‘tell me more about that’) to fit the flow of conversation with participants. (Page 9, Lines 188-9)

**Results**

**Reviewer #4:** To increase transferability of the study, please provide a description of the recruitment process (i.e. description of number of patients invited, declined - and reasons for declining).

**Author response:** Please refer to our response and changes to address a similar comment by Reviewer #1 on page 7 of this document.

**Reviewer #4:** The sample size seems to be realistic and sufficient to answer the research question. The sample consisted of a variability regarding sex, employment status, surgery type and caregiver status.

**Reviewer #4:** The result section is very comprehensive. The use of both Tables (Table 2 + 3) and text description leads to many repetitions. It is recommended to merge the sections, and to use citations in the texts, as this will lead to a more coherent results section.

**Author response:** We agree and have moved Tables 2 and 3 to Supplementary Tables and used citations in the text.

**Author change:** Please see S4 and S5 tables, and revised text section.

**Reviewer #4:** Line: 192 + 193. Suggest reporting only the numbers or the percentages.

**Author response:** We have reported as numbers in the revised manuscript.

**Author change:** ‘Mean (SD) age was 71 (10) years, 24 patients were female, and 24 patients had caregiver assistance. Twenty patients had a planned TKR, and 11 patients had a planned THR. One patient interviewed prior to a planned TKR cancelled surgery. Twenty-nine patients had a RAPT score of six or above. Of those that had the surgery, 13 patients received inpatient rehabilitation and the remaining 17 patients received rehabilitation-at-home, and none received hospital-at-home. (Page 11, Lines 258-263)

**Reviewer #4:** Table 2: Under theme “Patient unwillingness to seek help”. The citation “ I have a husband at home but he’s a busy person, I mean he will in around but the idea of him doing all that I do is unrealistic” does not really seem to support the theme about being unwilling to seek help.

**Author response/change:** We agree and have not used this quote in the revised S4 Table.

**Discussion**

**Reviewer #4:** The discussion includes a comprehensive summary of the findings, which is unnecessarily long. Instead, more critical discussion of the findings related to existing evidence is needed.

**Author response:** In the revised manuscript, we have provided a more concise summary of key findings in the first paragraph. In the second paragraph we have provided a more critical evaluation in relation to the existing evidence by comparing with relevant previous research that we referred to in the introduction which set up the rationale for doing the study.

**Author change:** ‘Fears around safety (e.g., being home alone), perceived lack of rehabilitation support and opportunity to rest at home, and patient unwillingness to seek help from caregivers emerged as major barriers to uptake of home-based care as perceived by privately insured THR and TKR patients. These barriers were commonly perceived by patients who received inpatient rehabilitation. Theoretical domains relating to these barriers included ‘emotion’, ‘environmental context and resources’ and ‘beliefs about consequences’. A live-in caregiver, positive perceptions of their home (e.g., freedoms) and fitness, caregiver willingness to support the patient’s choice, and advice from specialists emerged as major enablers to uptake of home-based care. Theoretical domains relating to these enablers included ‘beliefs about capabilities’, ‘skills’, ‘procedural knowledge’ and ‘social influences.’ (Paragraph 1 of Discussion, Page 25, Lines 541-50)

‘The key barrier and enabler themes identified in our study converged with themes identified in qualitative studies conducted in multiple countries, hospital settings (public and private) and with different aims (13-17). For example, a perceived lack of caregiver support and lack of confidence to cope with daily activities emerged as a barrier for home discharge in a study that examined patients’ choice of discharge destination in Singapore (16). ‘Paying for health insurance’ that emerged as a barrier to home-based care in our study is consistent with findings from a previous Australian study where patients’ perceived sense of entitlement influenced the discharge destination towards inpatient rehabilitation (17). However, most patients and caregivers in our study did not think financial factors influenced their preferred rehabilitation setting. It is possible that they were more focussed on their safety and the ‘optimal recovery’ than financial considerations. It is also possible patients and caregivers were not willing to divulge to the interviewer that financial factors influenced their preferred care.’ (Paragraph 2 of Discussion, Page 25, Lines 552-64)

**Reviewer #4:** Please provide discussion related to the transferability of the results, including application of results from a single-center private institution.

**Author response:** Our purpose was to provide a thick description of the participants, study context, and research process so that readers can judge if our findings from a private institution are transferable to their health systems. The insights provided in this study may be applicable to other Australian private hospitals with similar patient and clinical characteristics. We have clarified this in the discussion.

**Author change:** ‘Our findings from a single centre private hospital give valuable insight for health systems and providers designing suitable home programs that may be applicable to other Australian private hospitals with similar patient and clinical characteristics. We have also provided a thick description of the participants, study context, and research process so that readers can judge if our findings from a private institution are transferable to their setting. The role of the policy maker is to ensure financing, guidelines and objectives for home care services reduce demand for inpatient hospital beds, reduce costs and optimise health outcomes. The role of the clinician is to provide patients and caregivers with the knowledge, skills, resources, and confidence they require to engage in early discharge, hospital-at-home and rehabilitation-at-home. An understanding of our study findings may help to illuminate factors (i.e., barriers and enablers) influencing uptake of home-based care and provide health systems and clinicians with a framework for successful implementation. (Page 27, Lines 597-608)

**Reviewer comments from word file (to the editor).**

Thank you for giving me the opportunity to review this manuscript which is very interesting and covers an interesting subject. The aim of the study is valuable and important for the health system and providers designing home programs after TKR and THR. I have a few questions and suggestions for changes.

**Abstract Methods**

**Reviewer:** Please include the total number of semi-structured interviews

**Author change:** ‘Thirty-nine semi-structured qualitative interviews involving 31 patients….’ (Page 2, Line 33)

**Reviewer:** Please describe the participants characteristics e.g. mean age (SD), and % sex for both patients and caregivers

**Author change:** Thirty-nine semi-structured qualitative interviews involving 31 patients (mean age [SD] of 71 [10] years, 77% female) undergoing, or who have undergone, a TKR or THR at Cabrini Health, Melbourne, Australia and 14 of their caregivers (mean age [SD] of 69 [12] years, 57% female). (Page 2, Lines 33-6)

**Abstract Conclusion**

**Reviewer:** Typo: ”TKR or TKR” – one should be THR

**Author change: ‘…**for patients undergoing a TKR or THR in a private hospital setting.’ (Page 3, Line 61)

**Introduction**

**Reviewer:** Please state that the majority of joint replacements in Australia are performed in the private sector, as it can be different in other countries.

**Author change: ‘**The majority (70%) of joint replacements are performed in the private sector in Australia (11).’ (Page 4, Line 82)

**Methods**

**Reviewer:** Sampling - Please, can you elaborate the participant recruitment. How did you select the participants at the pre-admission information session– were they consecutively selected during the study period? Did you approach them one-by-one, or as a group?

**Author response:** Please refer to our response and changes to address a similar comment by Reviewer #1 on page 7 of this document.

**Reviewer:** Setting - Where did the interviews take place? Location, setting and was it a face-to-face interview? And was anyone else present during the interview**?**

**Author response:** We have clarified the data collection methods in the revised manuscript.

**Author change:** ‘Interviews were conducted face-to-face in participants homes or via the telephone, depending on participants preferences. Interviews were conducted one-to-one or in dyads (i.e., patient with their caregiver) depending on participant preferences.’ (Page 9, Lines 203-5)

**Reviewer:** Interview guide - How did you conduct the interview guide? (how did you come up with the questions) And did you adjust the research questions as you went along with the interviews? Or did you use the same questions for all interviews? And was the interview guide pilot tested prior to study commencement?

**Author response:** We have clarified the data collection methods in the revised manuscript.

**Author change: ‘**The interview guide was developed by our multidisciplinary team with clinical expertise in joint replacement surgery and rehabilitation, as well as academic expertise in qualitative and implementation research to ensure it was clinically relevant and comprehensible. The same interview questions were used for all interviews, and field notes were taken during and after interviews. The interview guide was not pilot tested prior to data collection.’ (Page 9, Lines 196-201)

**Reviewer:** Furthermore, were field notes made during/after interview?

**Author response:** Yes, field notes were taken during and after the interviews, and has been added to the revised manuscript.

**Author change: ‘**and field notes were taken during and after the interviews.’ (Page 9, Line 200)

**Reviewer:** Researcher characteristic - Please elaborate the personal characteristics of the interviewer. Clinical background/occupation, credentials, sex, years of experience within this field of research? Experience with qualitative research?

**Author response:** We have added the interviewer characteristics in the revised manuscript.

**Author change:** ‘All interviews were conducted by one male author (JW), an experienced physiotherapist (20 years) providing care at the hospital, who has in-depth knowledge of the hospital services and is a postdoctoral research fellow with experience in qualitative research. None of the participants were known to the interviewer.’ (Pages 9-10, Lines 208-11)

**Reviewer:** What was the participants knowledge of the interviewer? Did they know about the researcher? E.g., personal goals, reason for doing the study? The purpose of the study?

**Author response:** None of the participants were known to the interviewer but were provided with the following information about the study “The research project is aiming to gain an understanding of the potential obstacles, enablers and acceptability with alternative rehabilitation services to inpatient rehabilitation, such as home-based care”

**Author change:** ‘None of the participants were known to the interviewer.’ (Page 9-10, Lines 210-11)

**Author change: ‘**Interested participants were provided with the participant information and consent forms by the primary investigator. The primary investigator explained the purpose of the research was to gain an understanding of the potential obstacles, enablers and acceptability with alternative rehabilitation services to inpatient rehabilitation, such as home-based care. (Page 8, Lines 164-8)

**Results**

**Reviewer:** In the text it is stated: “one patient interviewed prior to a planned THR cancelled surgery”. However, in Table 1 it looks like it is a patient with TKR that is missing. See table 2, Under “interviewed before surgery/surgery type”. Please elaborate or modify accordingly.

**Author response:** We can confirm this was a typo in the text and has been changed to TKR.

**Author change:** ‘One patient interviewed prior to a planned TKR cancelled surgery’. (Page 12, Line 260-1)

**Reviewer:** Before describing the results, please sum up the number of barrier and enabler themes found, and the number of acceptability themes.

**Author change: ‘**S4 Table shows the eight themes mapped to theoretical domains, with illustrative quotes.’ (Page 14, Line 284)

**Author change:** ‘S5 Table 3 shows the five themes and illustrative quotes.’ (Page 21, Line 456)’

**Reviewer:** Please, provide more information in the text on the participants when describing barrier and enabler themes. In particular, I would like you to add after each quote whether the participant is a patient or caregiver, a female or a male, interviewed before or after surgery, RAH or inpatient – just like you have done in table 2. This is especially relevant if the quote is not listed in Table 2.

**Author response:** In the revised manuscript text, we have added after each quote whether the participant is a patient or caregiver (including caregiver type), a female or a male, interviewed before or after surgery, received rehabilitation-at-home or inpatient rehabilitation.

**Example of author change:** “Whatever she needed, I would look after her but, I didn’t have a limit or an expectation” (Caregiver 7, female, daughter, interviewed after surgery, received rehabilitation-at-home) (Page 16, Lines 321-3)

**Reviewer:** I´m a bit confused of how and when you use the quotation in the text under the barrier and enabler themes. Under the headline “Feeling unsafe versus feeling confident” I would like at least, one quote in the text that verifies that patients who received rehabilitation-at-home felt safe – I do not find Table 2 sufficient here.

**Author response:** In the revised manuscript text, we have added a supporting quote that verifies that patients who received rehabilitation-at-home felt safe. We have also amended the Table and included as Supplementary Table (S4 Table).

**Author change:** “My husband actually is a medical practitioner so that may have relieved any anxiety I might have had if I've gone home without having that support. I had that confidence nothing is going to go really wrong here.” (Patient 27, female, interviewed before surgery, received rehabilitation-at-home) (Page 15, Lines 311-4)

**Reviewer:** For the two following headlines the quotations in table 2 supports your text. Then, under the headline “Positive feelings about home over the hospital” you suddenly use the same quotation both in the text and in table 2.

**Author response:** In the original manuscript this duplication of results text and Table 2 was not intended. In the revised manuscript we have changed Table 2 to a Supplementary file (S4 Table).

**Reviewer:** Please align how and when you use the quotations in the text

**Author response:** In the revised manuscript we have included 1-2 illustrative quotes in the results text for each theme.

**Author change example:** “I need a rest - I'd like to be not cooking and I just want to switch off.” (Patient 22, female, interviewed before surgery, received inpatient rehabilitation) (Page 17, Lines 352-3)

**Reviewer:** Line 290: Typo (delete “patients and”) it is stated twice.

**Author response:** We have corrected this typo in the revised manuscript.

**Author change:** Some patients ~~and patients~~ and caregivers (Page 19, Line 399)

**Discussion**

**Reviewer:** Typo in the second line in the discussion section, “TKR or TKR” one should be THR**.**

**Author response:** We have corrected this typo in the revised manuscript.

**Author change: ‘**following a TKR and THR’ (Page 25, Lines 543-4)

**Reviewer:** What does the abbreviation “THA” and “TKA” stand for?

**Author response:** THA stands for total hip arthroplasty and TKA stands for total knee arthroplasty. These are typos and have been changed to THR and TKR (i.e., total knee and hip replacements).

**Reviewer:** In the discussion you state that there are many similar findings from other studies across the world. Please, elaborate the similarities/strengths to this study, and discuss the weaknesses in relation to other studies, discuss particularly any differences in results.

**Author response:** In the revised first and second paragraph of the discussion, we have been more explicit about how our findings compares to the other research in different settings worldwide, and a similar setting in Australia, with explanations of our results.

**Author change:** ‘The key barrier and enabler themes identified in our study converged with themes identified in qualitative studies conducted in multiple countries, hospital settings (public and private) and with different aims (13-17). For example, a perceived lack of caregiver support and lack of confidence to cope with daily activities emerged as a barrier for home discharge in a study that examined patients’ choice of discharge destination in Singapore (16). ‘Paying for health insurance’ that emerged as a barrier to home-based care in our study is consistent with findings from a previous Australian study where patients’ perceived sense of entitlement influenced the discharge destination towards inpatient rehabilitation (17). However, most patients and caregivers in our study did not think financial factors influenced their preferred rehabilitation setting. It is possible that they were more focussed on their safety and the ‘optimal recovery’ than financial considerations. It is also possible patients and caregivers were not willing to divulge to the interviewer that financial factors influenced their preferred care.’ (Paragraph 2 of Discussion, Page 25, Lines 552-64)

**Reviewer:** Please, do also discuss your findings regarding who chose to receive rehabilitation at home vs. inpatient rehabilitation (Table 1).

**Author response:** In the revised discussion, we have included an observation that the major barrier themes to home-based care were commonly perceived by patients who received inpatient rehabilitation.

**Author change:** ‘These barriers were commonly perceived by patients who received inpatient rehabilitation. (Page 25, Lines 544-5)

**Reviewer:** The participants who chose rehabilitation at home were younger, still employed, lived with a caregiver and had a higher RAPT-score. What does this mean, and what implication does it have for your clinical practice.

**Author response:** We think these characteristics could inform a more targeted approach when designing interventions likely to improve implementation of home-based care.

**Author change: ‘**They could be targeted to specific patients who prefer inpatient rehabilitation, as indicated by a lower RAPT score (i.e., 6 to 9), those living without a caregiver and retired.’ (Page 28, Lines 621-2)

**Reviewer:** Does the study leave any unanswered questions, and/or do you have any thoughts about future research questions/topics?

**Author response:** An important future research question we have included in the manuscript discussion is whether uptake of hospital-at-home and rehabilitation-at-home can be increased in private hospital settings by implementing patient-mediated interventions such as patient education or information materials. Another future question was to explore the views of administrators across multiple private hospitals and whether they see home-based care as beneficial.

**Conclusion**

**Reviewer:** I question whether you have the data to conclude that it is a patient behavioural change strategy that is needed to enable an increased uptake and acceptability of hospital-at-home and rehabilitation-at-home.

**Author response:** We agree this is an area of future research, and not an implication for practice.

**Author change:** Future research is needed to investigate strategies that dispel fears around safety and promote the benefits of home-based care for improving uptake of these services for patients undergoing a TKR or THR in a private hospital setting. (Page 29, Line 652)

**Reviewer:** In the discussion section, you sum up that it is components of care (pre-discharged home visits, information on recovery, access to health care support to ensure safe mobility and efficient exercise at home, and tailored domestic support) that is needed.

**Author response/change:** In the revised manuscript, this summary has been removed from the discussion, as the reviewer feedback suggested this was a repeat of the results.

**Reviewer:** The solution of a behavioural change strategy was introduced as a suggestion in the discussion section. Please elaborate or modify accordingly.

**Author response:** We have modified the discussion (and conclusion) by choosing not to discuss behavioural change techniques. Instead we have discussed more broadly that the findings can inform design of implementation strategies (e.g., education) by addressing the patient and caregiver perceived barriers and enablers to uptake of home-based care.

**Author change: ‘**Patient education and information interventions could ~~be used as behaviour change techniques to~~ target important theoretical domains…’ (Page 28, Line 616-7)
