# Supplementary material for: Barriers, enablers and acceptability of home-based care following elective total knee or hip replacement at a private hospital: A qualitative study of patient and caregiver perspectives
Source: PLoS One. 2022 Aug 24;17(8):e0273405. doi: 10.1371/journal.pone.0273405 (PMC9401137; doi:10.1371/journal.pone.0273405)
Supplement: Attachment — Submitted filename: Response to Reviewers.docx [file pone.0273405.s008.docx]

Carsten Bogh Juhl, PhD

Academic Editor

PLOS ONE

[cjuhl@health.sdu.dk](mailto:cjuhl@health.sdu.dk)

Re:	PONE-D-21-32322R1

Barriers, enablers and acceptability of home-based care following elective total knee or hip replacement at a private hospital: A qualitative study of patient and caregiver perspectives

Thank you for the opportunity to submit a revised version of the manuscript. We appreciate the reviewers’ comments and consider addressing these have led to an improved manuscript. Please find our response to the reviewers’ comments below. We have included the manuscript changes and used red type to highlight relevant changes to the manuscript text. We can also confirm the reference list is complete and correct.

**Reviewer #1**

Thank you for the opportunity to re-review your article. You have adequately addressed the comments I have raised in the previous round of review leading to an improved manuscript that I think is suitable for publication.

**Reviewer #2**

Thank you for the revised document. The previous raised comments have been taken into account.

**Reviewer #2:** P. 24, l. 525-526 and l. 536-537: Patients' data (patient, gender, time for interview and intervention) are listed differently than in the rest of the article.

**Author response:** We have corrected the formatting of the patients’ data to be consistent in the manuscript.

**Author change:** (Patient 9, female, 68 years, RAPT score 9, interviewed after surgery, received rehabilitation-at-home) (Page 25, Lines 557-8)

**Author change:** (Patient 12, female, 67 years, RAPT score 10, interviewed after surgery, received rehabilitation-at-home) (Page 26, Lines 572-4)

**Reviewer #3**

Thank you for letting me review this revised manuscript. The manuscript (especially the method section) is much more accurate and transparent in the description after the revision. However, I still have some minor issues to point out.

**Reviewer #3: Abstract**

According to the PLOS One author guideline the length of the abstract should not exceed 300 words – your abstract is 389 words. Please, revise this.

**Author response:** We have now revised the abstract length to 297 words. (Page 2, Lines 26-54)

**Reviewer #3: Methods**

**Researchers characteristics**

Please elaborate how much experience the interviewer has in qualitative research.

**Author response:** The interviewer is a lead author on three previously published qualitative studies, with experience conducting one-to-one interviews. Two examples are listed below.

Wallis JA et al. Perceptions about participation in a 12-week walking program for people with severe knee osteoarthritis: A qualitative analysis. Disability and Rehabilitation 2019. (Lead author and conducted one-to-one interviews interviews)

Wallis JA, et al Exploring views of orthopaedic surgeons, rheumatologists and general practitioners about osteoarthritis management. Musculoskeletal Care 2021 (Lead author, and directed the researcher who conducted the interviews)

**Author change:** ‘All interviews were conducted by a male postdoctoral research fellow (JW), an experienced physiotherapist (20 years) providing care at the hospital and with in-depth knowledge of the hospital services and experience in leading qualitative research, including conducting one-to-one interviews. (Pages 9-10, Lines 203-6)

**Reviewer #3: Results**

In the result section I would prefer that the author summed up/listed the barriers and enablers themes found, instead of listing them in a supplementary table (S4). Afterall, the barrier and enabler themes are the result of the study. The same goes for the acceptability themes – list the themes in the result section instead of in a supplementary table (S5). Listing the themes in the result section, may prevent the difference in themes listed in the abstract from those listed as headlines in the result section (they are not typed exactly in the same way) and they are not listed in the same order. This issue is also seen in the acceptability themes.

**Author response:** We have now listed the themes in the results section and amended the abstract to ensure the themes are presented the same way. We prefer to keep Supplementary Table 4 as this shows the barrier and enabler themes mapped to the Theoretical Domains Framework, with additional illustrative quotes. Supplementary Table 5 has been removed, with the illustrative quotes added into the results text.

**Author change:** Eight themes that emerged as barriers and enablers of home-based care included: feeling unsafe versus confident; caregivers’ willingness to provide support and patients’ unwillingness to seek help; less support and opportunity to rest; positive feelings about home over the hospital; certainty about anticipated recovery; trusting specialist advice over family and friends; length of hospital stay; paying for health insurance. A summary of the eight themes is included below, with illustrative quotes. S4 Table shows the themes mapped to theoretical domains, with additional illustrative quotes. (Page 14, Lines 281-7)

**Author change:** Five themes that emerged as acceptable components of home-based care included: home visits prior to discharge; specific information about recovery at home; one-to-one physiotherapy and occupational therapy perceived as first-line care; medical, nursing and a 24/7 direct-line perceived as second-line care for complications; no one size fits all model for domestic support. A summary of the five themes and illustrative quotes is included below. (Page 22, Lines 468-73)

**Reviewer #3:** After each quotation it would have been nice also to know the participant´s age and RAPT score, to get a quick impression of the participant´s mobility

**Author response and changes:** For each of the patients’ quotes included in the results text, we have now included their age and RAPT score. For the caregivers, we have also included their age. Two examples are shown below.

“(Patient 27, female, 65 years, RAPT score 12, interviewed before surgery, received rehabilitation-at-home)” (Page 16, Lines 317-8)

“(Caregiver 13, male, spouse, 75 years, interviewed after surgery, patient received inpatient rehabilitation)” (Page 25, Lines 545-6)

**Reviewer #3:** In the last two quotations under the headline “paying for health insurance” what kind of rehabilitation did these patients receive?

**Author response:** For caregiver 3, the patient had received inpatient rehabilitation. For caregiver 5, the patient had received rehabilitation-at-home. These details have been added to the results.

**Author change:** “He has private insurance and all covered. Why to send him home?” (Caregiver 3, female, paid caregiver, 55 years, interviewed before surgery, patient received inpatient rehabilitation) (Page 21, Lines 455-7)

**Author change:** “We’re in a position where we’re fortunate where we can pay [for home-based care] now.” (Caregiver 5, female, sibling, 73 years, interviewed before surgery, patient received rehabilitation-at-home) (Page 22, Lines 463-5)

**Reviewer #3:** Under the headline “no size fits all model for domestic support” what does P9 stand for? And please do also elaborate (P12, F, A, RAH).

**Author response:** P9 stands for Patient 9. We have elaborated on these results in the revised manuscript.

**Author change:** (Patient 9, female, 68 years, RAPT score 9, interviewed after surgery, received rehabilitation-at-home) (Page 25, Lines 557-8)

**Reviewer #3: Discussion**

In the discussion section (line 571-579), where you discuss the contrast between patients´ unwillingness to seek help from caregivers and caregivers willingness to support the patients. In this path you refer to a study (ref 15) is this a comparison with your study? – if so please elaborate the finding of this study (ref 15) and compare them to your findings and e.g. state if it is an enabler or barrier to home-based care/rehabilitation.

**Author response:** Reference 15 had examined the experiences of live-in caregivers (spouses and offspring) in looking after patients following early discharge to hospital-at-home following a joint replacement. This study was not a direct comparison with our study findings that examined the barriers and enablers to home-based care. We had cited this study to show the caregiver’s role may be relatively small. Therefore reference 15’s finding (caregiver’s role) combined with our findings (caregivers’ willingness to support the patient’s choice) may indicate an important enabler for home-based care. In the revised manuscript, we have elaborated on reference 15’s aim and how it may support our findings.

**Author change:** Another unique finding was caregivers’ willingness to support the patient’s choice for rehabilitation-at-home or inpatient rehabilitation, and to provide support to patients during rehabilitation-at-home. This contrasted with patients’ unwillingness to seek help from caregivers in our study. A previous qualitative study that examined the experiences of live-in caregivers (spouses and offspring) in looking after patients during hospital-at-home following a joint replacement has shown that caregivers may provide a small degree of assistance with hygiene, dressing, mobility and overall responsibility (15). A review of hospital-of-home has also shown a low level of burden experienced by caregivers after joint replacement (7). Our findings suggest that clinicians should encourage their patients to ask family or friends to be their caregiver in the early days at home and this may be an important enabler to home-based care following a THR or TKR. As well as providing assurance to the patient about the low level of burden (7) and small degree of assistance their caregivers may need to provide (15). (Pages 27-28, Lines 607-19)

**Reviewer #3: Conclusions**

In your conclusion, could you please be more specific about what is “multiple factors”?

What do you want your readers to remember from reading this paper? Any specific factors?

Please remember to align this with the conclusion in the abstract.

**Author response:** We have amended the conclusions in the manuscript and abstract by including examples of key factors.

**Author change:** Manuscript conclusion: Multiple factors emerged, such as feeling unsafe and caregivers’ willingness to provide support, that influence implementation of home-based care from the perspectives of privately insured patients and caregivers. Our findings give valuable insight for health systems and providers designing suitable home programs. Future research is needed to investigate strategies that dispel fears around safety and promote the benefits of home-based care for improving uptake of these services for patients undergoing a TKR or THR in a private hospital setting. (Page 31, Lines 690-6)

**Author change:** Abstract conclusion: Multiple factors, such as feeling unsafe and caregivers’ willingness to provide support, may influence implementation of home-based care from the perspectives of privately insured patients and caregivers. Our findings provide insights to inform design of suitable home-based care following joint replacement in a private setting. (Page 3, Lines 51-4)

**Reviewer #4**

Thank you for the opportunity to review the article again. The authors have answered all my questions and comments, and have satisfactorily made appropriate changes to the manuscript.

I have only a few suggestions for correcting potential errors:

**Reviewer #4:** Please check the wording in the following line. Page 8, lines 185: "we invited participation in the study at consecutive group-based, face-to-face preadmission information sessions, conducted weekly at the hospital".

**Author response:** We have amended the sentence as below which we believe has improved the writing.

**Author change:** “We invited participation in the study at consecutive preadmission information sessions. These group-based sessions were conducted weekly at the hospital and face-to-face. (Page 8, Lines 155-6)

**Reviewer #4:** Page 10, lines 237-239: participants x 3 (written as "patients" in all other places).

**Author response and change:** We have changed “participants” to “patients and caregivers” throughout the manuscript to improve consistency of the writing.
